# Bibliometric Indicators of Russian Journals by JCR-Science Edition, 1995-2010

**Published:** 2013

**Authors:** A.N. Libkind, V.A. Markusova, L.E. Mindeli

**Affiliations:** All-Russian Institute for Scientific and Technical Information, Russian Academy of Sciences, Usievicha Str., 20, Moscow, Russia, 125190; Institute for the Study of Science, Russian Academy of Sciences, Butlerova Str., 12, p/o box 6, Moscow, Russia, 117485

**Keywords:** article, impact factor, Russian journal, full data set, bibliometric indicators, expected citation rate, JCR

## Abstract

A representative empirical bibliometric analysis of Russian journals included in the Journal Citation Reports-Science Edition (JCR-SE) for the time period 1995–2010 was conducted at the macro level (excluding the subject categories). It was found that the growth in the number of articles covered by JCR (a 1.8-fold increase compared to 1995) is ahead of the growth rates of Russian publications (1.2-fold increase). Hence, the share of Russian articles covered by JCR-SE was down from 2.5% in 1995 to 1.7% in 2010. It was determined that the number of articles published in an average Russian journal reduced by 20% as compared to the number of articles in an average journal of the full data set. These facts could partly shed light on the question why Russian research performance is staggering (approximately 30,000 articles per year), although the coverage of Russian journals has expanded to 150 titles. Over the past 15 years, a twofold increase in the impact factor of the Russian journals has been observed, which is higher than that for the full data set of journals (a 1.4-fold increase). Measures to improve the quality of Russian journals are proposed.

## INTRODUCTION

It is a well-known fact that the development of scientometrics was triggered by the Science Citation Index (SCI) creation by Dr. E. Garfield of the Institute for Scientific Information (ISI)1 in 1964. This event has become a revolutionary factor not only for the development of a novel unique information tool, but it has also led to the development of a new scientific discipline, scientometrics (or more commonly known today as bibliometrics). According to Dr. E. Garfield, “we are witnessing the transformation of bibliometric studies into a field of industry: the assessment of the output of the research carried out by universities and by scientific teams” [1]. Although there has been growing dissatisfaction among members of the scientific community with* 1 K2001Kg. This institute belongs to the ThomsonReuters (TR) company.* the passion of bureaucrats from various foundations and ministries for all kinds of ratings and evaluations, the effect of these indicators on the level of funding for basic research is becoming greater.

With the accumulation of a large collection of bibliographic data at the ISI and the simultaneous rapid development of computer technology in the United States, a new information product was developed based on the interrelationships between the journals the Journal Citation Reports (JCR ), which is published annually. Since 1978, JCR Social Sciences Edition has been published. Beginning from 2009, both editions of the JCR have been available online as part of the Web of Knowledge (WoK). JCR shows the relationship between citing and cited journals and contains data on a journal’s impact factor (IF). The concept of impact factor was proposed by Dr. E. Garfield in collaboration with Dr. Irving Sher in 1955 [2]. The introduction of this term contributed to an increase in the quality of libraries’ acquisition. However, IF quickly became popular as a symbol of the scientific prestige of a journal, although its value significantly varies depending on the field of science and its relevance to the subject field. As Dr. Garfield has mentioned, “Many scholars and editors are currently making a terrible mistake thinking that SCI was created exclusively to produce its by-product, JCR . The major purpose of these resources was not only to aid information retrieval but also to use it as an alerting tool, i.e., for selective dissemination of information.” [1]

One can say it is a bulk of literature considering the drawbacks and shortages of the IF, but it is impossible to receive a research grant at foreign universities, unless the grant applicant has publica tions in journals with a high IF. It is impossible to list the number of bibliometric studies based on the use of JCR statistics and devoted to various aspects of IF application, including the analysis of growth rates in the scientific literature by a specific subject category, to the factors affecting the IF value within subject categories, as well as to the use of IF by funding organizations as a measure to assess scientific activity at the level of university departments and research groups.

In certain field of science, excessive passion for using the IF as an indicator of the research activity efficiency and decision-making regarding promotion within organizations (getting tenure), as well as for assessing the viability of faculties and colleges, results in negative consequences. Thus, it was noted in [3] that “the desire by research personnel at medical colleges in many countries to be published exclusively in journals with a high IF threatens the very existence of medical nursing journals and causes cessation of the publication of books and chapters in books for which the IF values are not calculated.” In the Netherlands, the desire to be published exclusively in the journals listed in the JCR has led to cessation of the publication of Dutch journals on social sciences [4]. A significant contribution to the development of bibliometric studies related to the normalization of the IF in various fields of science has been made by Braun T. [5], Glanzel W., [6], and Leydesdorff L. [7].

The increasing amount of scientific literature and transformation of the industrial society into the knowledge economy have resulted in an expansion of the journals’ coverage by JCR . While the first JCR edition contained statistics on 3,000 journals, the number of journals in the JCR -Science Edition increased up to 8,700 in 2010. According to German specialists [8], the reason behind the expansion of journals coverage processed by ThomsonReuters is due to its competition with Elsevier, which has been issuing the Scopus since 2005. Scopus is similar to SCI to a significant extent. It consists of over 18,000 journals. The criteria for the selection of journals to be included in the Thomson Information Resources were thoroughly discussed in [9]. The IF depends on the language of publication, research field, and sociocultural traditions of science. The first analysis of Soviet journals was performed by Dr. E. Garfield [10]. I. Marshakova’s article devoted to a comparative analysis of the IF of Russian and Polish journals on mathematics should be mentioned among the papers that discussed the analysis of Russian journals IF [11].

No representative in-depth analysis of the bibliometric indicators of Russian journals has been performed within the past 20 years. The aim of this empirical study carried out at the macro level (regardless of the journal’s subject category) was to identify the trends in the bibliometric indicators of Russian journals and to compare them to the global trends by analyzing the annual sets of the JCR -Science Edition (JCR -SE) for the time period 1995–2010. Since only four Russian journals have been included in the JCR -Social Science Edition during the past decade, the data on the comparative analysis of these journals against the trends in the full data set would not be statistically significant.

The choice of the subject of our research is directly related to the reform of the basic research and the higher education sectors being carried out in Russia. This reform is accompanied by growing attention by the President and the Government of the Russian Federation to bibliometric indicators as a tool for research efficiency evaluation. On May 7, 2012, Russian President Vladimir Putin signed a decree on “Measures for the Implementation of the State Policy in the Field of Education and Science.” This decree, in particular, contains provisions regarding increasing competition among Russian universities. In accordance with the latter, “at least five Russian universities must be included in the list of the top 100 best universities in the world by 2020, according to one of three World University Rankings.” [12] Accomplishing this task is largely associated with the IF of the journals in which the articles of the teachers of the higher education sector will be published. Our earlier studies have demonstrated that ~ 60% of Russian articles included in the Web of Science (WoS) were published in Russian journals [13]. Meanwhile, despite the expansion of Russian journals coverage by Web of Science, the number of Russian publications over recent years has remained stable and does not exceed 30,000 articles. Significant financial investments by the Russian Government in universities have resulted in the fact that universities pay authors a substantial amount of money for publication in journals covered by WoS. Furthermore, the amount of compensation depends on the IF value.

## METHODS

Issues of the JCR -SE for the time period 1995–2010 were used as the source of bibliometric statistics data. During the period from 1995 to 2008, JCR was issued on CD-ROM, and since 2009 it has become available as a part of Web of Knowledge via the Internet. Unfortunately, the CD-ROM for 2001 was not available; therefore, the statistics were collected only for a 15-year period: 1995–2000 and 2002–2010.

Bibliometric statistics were collected from the JCR for each year for the following indicators: 

• the number of Russian journals and the total number of journals; 

• share of Russian journals in JCR ; 

• the annual number of articles in an average Russian journal and in an average journal of the full data set; 

• the average number of articles published in a Russian journal and in an average journal of the full data set;

• the average IF of a Russian journal and the average IF of a journal of the full data set

• the expected response for a Russian journal.

1 To calculate the expected response (ER ) to articles published in a specified journal (either Russian or one in the full data set) for a given year was estimated. The expected response is the number of articles published in the journal in a given year *t *multiplied by the IF of this journal over that year *t*:

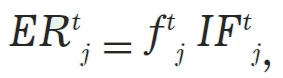



where *f^t^_j_* is the number of articles published by the journal*_j_* over the year *^t^*; *IF^t^_j_* is the IF of the journal *_j_* in the year *^t^*; and *ER^t^_j_* is the expected response to articles published in the journal *_j_* over the year *^t^*.

*L_t_* is the list of journals (Russian or total journals set) in a given year *^t^*. The total estimated response* S_ER^t^*to articles published in all journals from the list *L_t_*over the year *^t^* was calculated according to the formula

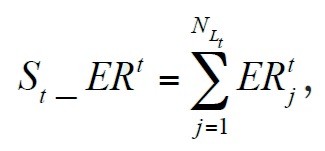



where *N_Lt_* is the total number of journals in the list *L_t_*.

The original information contained in each annual set of JCR -SE was uploaded into a special database based on the MS SQL Server 5. As a result, statistics were collected for each of the aforementioned indicators during each of the 15 years.

## RESULTS AND DISCUSSION

**Fig. 1:**
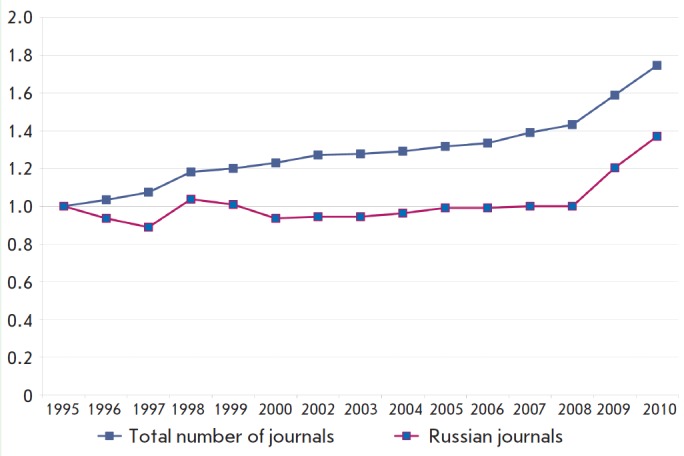
Growth rate of the number of Russian journals and the total number of journals covered by JCR-SE as compared to that in 1995

The results of our study indicate that there is a steady increase in the number of journals indexed in WoS^2^, and, correspondingly, JCR : 4,655 titles were covered by JCR - SE in 1995, and 8,073 titles were covered in 2010. In other words, there has been a 1.75-fold increase. In 1995, this database covered 108 Russian journals; this number increased to 148 titles in 2010; i.e., a 1.37-fold increase was observed (*Fig. 1*). The difference between the growth rates of the Russian and the full data set has a negative impact on Russian research output. *Figure 1 *shows diagrams characterizing the growth rates of the Russian journals and the full data set.

A significant decline was observed in 1997 (96 titles). In 1998, the number of journals increased to 112. The relatively stable number of titles during the period between 2004 and 2008 rose to 130 and 148 in 2009 and 2010, respectively. The changes in the number of Russian journals during the studied period were partially associated with ThomsonReuters processing changes: from processing Russianlanguage versions to English-language ones. The increase in the coverage of the Russian journals is related both to the improvement in the quality of Russian journals and competition with the Scopus, which covers 230 Russian titles.

Our data regarding the growth of scientific literature virtually coincide with the data [7] obtained from WoS statistics (both versions of the SCI-Expanded and SSCI-Expanded) for the period 2000–2008. According to this publication, the number of journals has increased by 29%, with an average growth rate of 3.3%. The highest growth rate was recorded in 2007–2008. It is a known fact that a journal must have been listed in WoS for at least two years before it receives an IF. Hence, the IFs of journals published in 2008 could appear in the JCR only in 2010.

The growth in the global literature is attributed to several factors, including the development and globalization of science, emergence of a new research area, and appearance of new journals, as well as ThomsonReuters policy to expand journals coverage in WoS. As mentioned in [12], the number of publications in WoS is growing rapidly. Our results have shown that JCR -SE covered 607,049 articles in 1995, while the number of articles reached 1,080,209 in 2010 (i.e., a 1.78-fold increase was observed). The number of Russian articles covered by JCR -SE has also increased; however, only a 1.22-fold increase was observed. The proportion of Russian articles among the total number of publications covered by JCR -SE was down from 2.48% in 1995 to 1.7% in 2010.

**Fig. 2:**
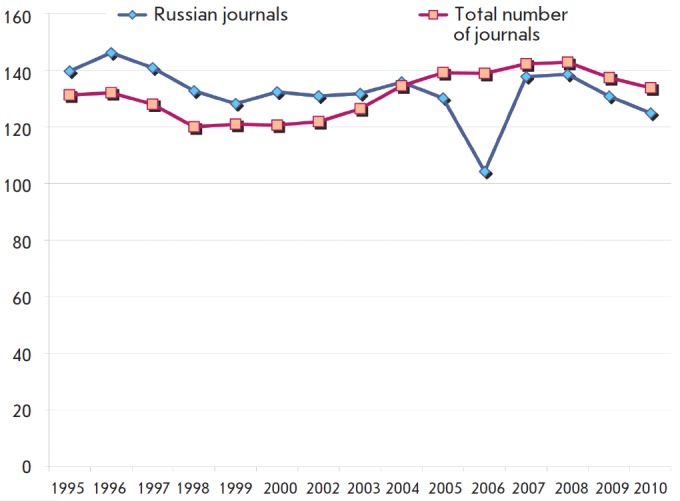
Changes in the number of articles per single average Russian journal and per journal among the total number of journals covered by JCR-SE

Our goal was to find out to what extent the increase in the total number of Russian articles is associated with the expansion in the journal coverage by JCR -SE or whether the growth is attributed just to the increase in the number of articles per single average journal. So we investigated the trends of a single average Russian journal and a journal from the total journals’ set over the studied period. *Fig. 2*shows two diagrams characterizing the trends of both types of journals.

The data presented in *Fig. 2* demonstrate the “ups” and “downs” in the average number of articles per single Russian journal. The sharp fall shown in the diagram for 2006 is obviously associated with the changes that were occurring at that time at the Nauka publishing house and at the company respon sible for the translation of Russian journals. Over all, the number of articles per single average Russian journal fell by 18.3% (or 20 articles) as compared to 1996. An opposite trend was evident in the total journals set: a 1.9% growth rate was observed. A conclusion can be drawn that the significant decrease in the number of articles (by almost 20%) per single Russian journal that has occurred over the past 15 years is one of the main reasons for the lack of growth in Russian research output.

In order to assess all the causes of the stagnation in the number of Russian publications, one needs to collect statistics related to all the articles of Russian researchers published in foreign journals. We compared the number of Russian publications covered by WoS to that covered by JCR -SE between 2006 and 2010. These data are listed in Table 1.

**Table 1:** Share of articles published in Russian journals, %

Year	Share of articles published in Russian journals, %
2006	44.6
2007	44.3
2008	43.7
2009	45.8
2010	53.6

It is clear from Table 1 that the number of articles published in Russian journals increased significantly during the period 2009–2010. The share of Russian publications in foreign journals remains high. This demonstrates that Russian science remains part of global science.

**Fig. 3:**
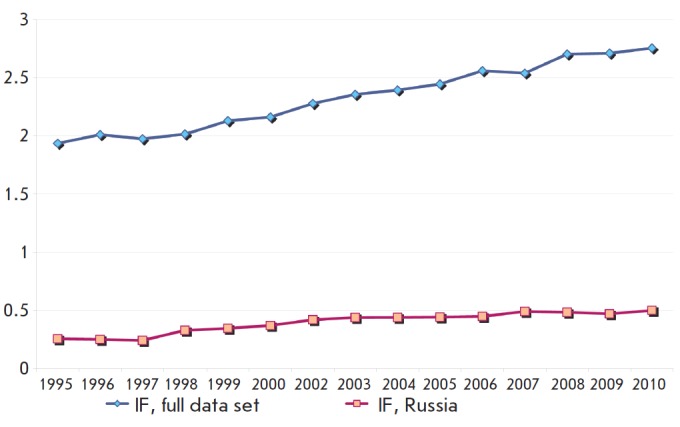
Changes in the number of articles per single average Russian journal and per journal among the total number of journals covered by JCR-SE

A journal's IF plays an important role in evaluating a scientist’s performance (however, too much significance is often attached to it). In 2005, the average IF of a single Russian journal was 0.27 compared to 1.3 IF for a journal in the full data set and that of a journal among the full data set was 1.3; i.e., there was a 4.8-fold difference. During the survey period, a tendency toward increased IF values was observed for journals in both groups. These data are presented in *Fig. 3*.

The average IF of a Russian journal for the studied period has increased 1.75-fold, although it still remains relatively low. However, despite the obvious difference in the IF values between the two groups of foreign (http://urfu/fileadmin/ user_upload/docs/science/Prikaz_ 122_2013.pdf).

The journals assigned to a particular subject category and ranked by the highest value of the IF, which account for the first 25%, are considered to be the most important ones. This research has resulted in the widespread application of the 25% subject category journals both abroad and in Russia. For instance, the lecturers at Moscow State University receive an additional monthly financial reward for the publication of articles in the international journals included in the top 25% of subject category according to the Web of Science classification http://istina.imec.msu/statistics/journals/top.

As we have mentioned above, the IF strongly depends on the subject category, since the citation rate between immunology and mathematics differs by almost an order of magnitude. The list of Russian journals in the JCR -SE for 2010 contains only nine journals with IF higher than one. However, only a few of them have been listed in the top 25 or 50% most prestigious journals in the relevant subject category. *Table 2*contains data on these journals. It is noteworthy that among eight journals of this category, three are mathematics journals with IFs substantially lower than one.

**Table 2:** Russian journals included in the top 25 and 50% of the corresponding subject category

Journal	Impact factor	Rank among the Russian journals with respect to IF	Rank in the JCR subject category	Subject category	Number of journals in the subject category	The size of the share in the JCR subject category, %
Laser Physics	1.362	5	34	Optics	79	50
Physics-Uspekhi	2.245	2	18	Interdisciplinary sciences, physics	80	25
Russian Chemistry Review	2.346	1	43	Interdisciplinary sciences, chemistry	154	50
JET P Letters	1.557	3	32	Interdisciplinary sciences, physics	80	50
Functional Analysis and Its Applications	0.688	25	103	Mathematics	279	50
Moscow Mathematical Journal	0.721	21	93	Mathematics	279	30
Journal of Mathematical Physics	1.131	6	28	Interdisciplinary sciences, physics	55	50
Journal of Experimental and Theoretical Physics	0.946	12	41	Interdisciplinary sciences, physics	80	50

A number of Russian journals with relatively high IFs were not included even in the top 75% of journals. This applies to *Biochemistry*, which ranks fourth among Russian journals, with an IF of 1.402. This journal belongs to the “Biochemistry and molecular biology” subject category, consisting of 286 journals.* Biochemistry *in this subject category ranks 234^th^, and it was not even included in the top 75%. Another example is *Astronomy Letters*, with an IF value of 1.091, which ranks 8^th^ among Russian journals with respect to the IF and 36^th^ among 55 journals in the “Astronomy and astrophysics” subject category. This journal belongs to the 75% group. The remaining Russian journals have relatively low rankings in their relevant subject categories. We consider it necessary to bolster the credibility of Russian journals in the eyes of the international scientific community. It would be reasonable to invite foreign colleagues to join editorial boards and to provide each issue of the journal with content in English. The authors have to provide an abstract and a list of keywords in the Russian and English languages. The articles should to be accompanied by a list of references. The establishment of language counseling centers for assistance in editing the articles written by Russian authors in the English language seems relevant. Publications by leading scientists in Russian journals contribute to the development of Russian science and could guide young researchers in their scientific endeavors. Since the Ministry of Education and Science of the Russian Federation attaches great importance to the bibliometric indicators of Russian science, it could use the experience of foreign universities and colleges, which have special training courses that teach how to prepare articles and reports, technical papers, and grant applications.

The expected response (*ER*) to journal articles is the number of articles multiplied by the journal IF in a specific year. In order to calculate the expected response of the aggregate of Russian journals (or that of the full data set), the sum of these products throughout the entire given set (Russian journals or the full data set) for the year under study is used. It can be noted that a maximum expected response is observed for the articles published in Russian journals twelve years ago. The falls on the curve are associated with the technological changes in the journals’ processing as has been mentioned above.

It should be mentioned that these response data only apply to Russian articles published in Russian journals. Since over 45% of Russian articles are published in foreign journals, the total response to Russian articles is much higher.

## CONCLUSIONS

A representative empirical bibliometric analysis of the Russian journals covered by Journal Citation Reports-Science Edition (JCR -SE) for the period 1995–2010 has been conducted for the first time at the macro level (excluding subject categories).

The growth of the total number of articles (1.8-fold increase) as compared to 1995 outpaces the growth rates of Russian publications (1.2- fold increase). As a result, the share of Russian articles among the total number of publications decreased from 2.49% in 1995 to 1.71% in 2010.

It was determined that the number of journals among the full data set and that of Russian journals increased by factors of 1.75 and 1.37, respectively.

Over a 15-year period, the number of articles published in a single average Russian journal has decreased by 17.9% as compared to that in 1995. An increase by 1.9% was observed for the full data set. This fall is the reason behind the stagnation in the Russian research output for the period 2008–2010, despite the increase in the number of Russian journals covered by Web of Science. Another cause is the decline in the share of Russian journals in the total number of journals covered by Web of Science from 2.34% in 1995 to 1.83% in 2010.

Despite the fact that the weighted average IF of Russian journals remains significantly lower than that of the journals among the total number of journals, it increased 1.96-fold as compared to that in 1995. Meanwhile, the weighted average IF of the full data set is characterized by a 1.42-fold increase.

Our data provide reliable statistics for policy makers and editorial boards. In order to improve the bibliometric indicators of Russian science, it is necessary to improve the quality of Russian journals translation into English. Furthermore, a program for graduate and undergraduate students on “How to prepare a research paper” is required. The Ministry of Education and Science of the Russian Federation should play a key role in this preparation.
